# Long-term risk of primary liver cancers in entecavir versus tenofovir treatment for chronic hepatitis B

**DOI:** 10.1038/s41598-020-80523-7

**Published:** 2021-01-14

**Authors:** Te-Sheng Chang, Yao-Hsu Yang, Wei-Ming Chen, Chien-Heng Shen, Shui-Yi Tung, Chih-Wei Yen, Yung-Yu Hsieh, Chuan-Pin Lee, Meng-Ling Tsai, Chao-Hung Hung, Sheng-Nan Lu

**Affiliations:** 1 Division of Hepatogastroenterology, Department of Internal Medicine, Chiayi Chang Gung Memorial Hospital, Chiayi, Taiwan; 2 Department of Traditional Chinese Medicine, Chiayi Chang Gung Memorial Hospital, Chiayi, Taiwan; 3 Health Information and Epidemiology Laboratory, Chang Gung Memorial Hospital, Chiayi, Taiwan; 4 Division of Hepatogastroenterology, Department of Internal Medicine, Kaohsiung Chang Gung Memorial Hospital and Chang Gung University College of Medicine, Kaohsiung, Taiwan

**Keywords:** Gastroenterology, Oncology

## Abstract

It remains controversial whether entecavir (ETV) and tenofovir disoproxil fumarate (TDF) is associated with different clinical outcomes for chronic hepatitis B (CHB). This study aimed to compare the long-term risk of ETV versus TDF on hepatocellular carcinoma (HCC) and intrahepatic cholangiocarcinoma (ICC) in CHB patients from a large multi-institutional database in Taiwan. From 2011 to 2018, a total of 21,222 CHB patients receiving ETV or TDF were screened for eligibility. Patients with coinfection, preexisting cancer and less than 6 months of follow-up were excluded. Finally, 7248 patients (5348 and 1900 in the ETV and TDF groups, respectively) were linked to the National Cancer Registry database for the development of HCC or ICC. Propensity score matching (PSM) (2:1) analysis was used to adjust for baseline differences. The HCC incidence between two groups was not different in the entire population (hazard ratio [HR] 0.82; 95% confidence interval [CI] 0.66–1.02, p = 0.078) and in the PSM population (HR 0.83; 95% CI 0.65–1.06, p = 0.129). Among decompensated cirrhotic patients, a lower risk of HCC was observed in TDF group than in ETV group (HR 0.54; 95% CI 0.30–0.98, p = 0.043, PSM model). There were no differences between ETV and TDF groups in the ICC incidence (HR 1.84; 95% CI 0.54–6.29, p = 0.330 in the entire population and HR 1.04; 95% CI 0.31–3.52, p = 0.954 in the PSM population, respectively). In conclusion, treatment with ETV and TDF showed a comparable long-term risk of HCC and ICC in CHB patients.

## Introduction

Chronic hepatitis B (CHB) is one of the most common chronic viral infections worldwide, affecting approximately 350 million people^1^. It may result in serious complications, such as liver failure, advanced cirrhosis, and/or hepatocellular carcinoma (HCC) among 15–40% of infected patients^1–3^. The risk of cirrhosis and/or HCC in CHB increases proportionally as serum HBV DNA levels increase^3^. Previous studies have demonstrated that antiviral treatment with nucleos(t)ide analogues (NA) treatment, either by lamivudine, entecavir (ETV) or tenofovir disoproxil fumarate (TDF), can reduce the risk of HCC development, cirrhotic events and mortality^4–8^.

Currently, both ETV and TDF are equally recommended as first-line NAs treatment for CHB patients. ETV and TDF are high potent and high genetic barrier NAs, with excellent antiviral activity in both hepatitis B e antigen (HBeAg)-positive and HBeAg-negative CHB. Nevertheless, a recent study from Choi et al. showed that TDF treatment was more effective in lowering risk of HCC compared with ETV treatment in a Korean nationwide cohort of CHB patients^9^. This result was also successfully validated from a hospital-based cohort^9^. However, subsequent studies did not show the consistent results even though propensity score (PS) matching and inverse probability of treatment weighting analysis was used to minimize selection bias^9–13^. A multi-center cohort study in South Korea demonstrated that the overall HCC incidence was not statistically different between ETV and TDF treatment^10^. More recently, TDF treatment was also not associated with a lower risk of HCC than ETV treatment among Asian and non-Asian patients in a multi-center study^12^.

Intrahepatic cholangiocarcinoma (ICC) is the second most common type of primary liver cancer behind HCC, accounting for 10–15% of primary liver cancers^14^. The incidence and mortality of ICC is markedly increasing over the past two decades worldwide^15–17^. In recent years, several studies have indicated that chronic HBV infection is a significant risk factor for ICC^17–19^. HBV-associated ICC patients displays significantly different clinicopathological characteristics as well as survival outcomes^17–19^. A recent study has demonstrated that preoperative NAs therapy could decrease HBV reactivation and prolong long-term survival after liver resection for ICC patients with a high HBV DNA level^20^. To our knowledge, there were no studies to evaluated the ICC incidence in patients treated with ETV vs. TDF.

We therefore conducted a retrospective cohort study using the Chang Gung Research Database (CGRD) for CHB patients undergoing ETV or TDF treatment since 2011. CGRD is a de-identified database derived from medical records of Chang Gung Memorial Hospital (CGMH), which was the largest multi-institutional electronic medical records collection in Taiwan^21^. CGMH is currently the largest Taiwanese medical care system, comprising 4 tertiary‐care medical centers and 3 major teaching hospitals. This medical care system, with more than 10,000 beds and over 280,000 inpatients per year, provides about 10% of all medical service used by the Taiwanese people annually^21^.

## Materials and methods

### Study cohorts

Between January 2011 and October 2018, a total of 21,222 CHB patients who were initially treated with ETV or TDF were screened for eligibility. Patients who had co-infection with human immunodeficiency virus or hepatitis C virus by serological assays or preexisting cancer were excluded. Also, patients with follow-up duration and exposure to ETV or TDF of less than 6 months, HCC or ICC development during the first 6 months, and missing data of serum levels of alanine aminotransferase (ALT), aspartate aminotransferase (AST), platelet counts, alpha-fetoprotein protein (AFP), albumin, bilirubin, international normalized ratio (INR) or exposure to both ETV and TDF during the follow-up period were excluded. Finally, 7248 patients (ETV: 5348, TDF: 1900) were included in the analyses. We collected the date of NAs prescription and the number of days supplied. The defined daily doses (DDDs) recommended by the WHO (World Health Organization)^22^ were used to quantify the usage of ETV or TDF. Cumulative DDD was estimated as the sum of dispensed DDD of ETV or TDF from the starting date.

Liver cirrhosis was either histopathologically (n = 65) or clinically diagnosed (n = 2115). Clinical diagnosis was based on the ultrasound findings as coarse liver parenchyma with nodular contour and small liver size and the presence of features of portal hypertension^23,24^. The Child–Turcotte–Pugh (CTP) score was calculated to classify cirrhosis, and those with a CTP score above 6 (class B or C) were defined as having hepatic decompensation. The endpoints of this study were HCC or ICC, which were identified based on the diagnosis codes retrieved from Cancer Registry Database and medical records in CGRD. The diagnosis of HCC or ICC was ascertained by histology or imaging criteria compatible with the guidelines of the American Association for the Study of Liver Disease^25^. In clinical principle, patients with combined HCC and ICC were classified as ICC. Informed consent was obtained from all participants. This study was approved by the Research Ethics Committee of Chang Gung Memorial Hospital (IRB no. 201901734B0C501) and was conducted in accordance with the principles of Declaration of Helsinki and the International Conference on Harmonization for Good Clinical Practice.

### Matched cohort

To further examine the effect of NAs use, we used PS to estimate the probabilities of assigning a patient to use ETV or TDF, given background variables including age, sex, ALT, AST, platelet counts, AFP, albumin, bilirubin, INR, AST to platelet ratio index (APRI), fibrosis index based on 4 factors (FIB-4), cirrhosis, diabetes, CTP score, and Charlson Comorbidity Index (CCI). ETV and TDF patients were matched by using PS at a ratio of 2:1. Overall, 4956 patients (1652 matched sets) were included in the matched cohort. Serum concentrations of albumin, INR of prothrombin time, AFP, platelet count, liver cirrhosis, diabetes, FIB-4, APRI, CTP score and CCI were regarded as potential confounders and identified form medical records. Besides, previous exposure to lamivudine, telbivudine or adefovir was identified as a confounder.

### Statistical analyses

The distribution of demographic factors, laboratory data and the proportions of co-morbidities between the ETV and TDF patients in the study cohort and matched cohort were compared. We used the Kaplan–Meier method to estimate the cumulative incidences of HCC and ICC. The log-rank test was performed to examine differences in the risk for HCC and ICC in the cohort. Finally, Cox proportional hazards models were used to compute the hazard ratios (HRs) accompanying 95% confidence interval (CI) after adjustment for potential confounders. Two-tailed *p* < 0.05 was considered to be significant.

Patients with a death date in the admission file and those from the beneficiaries register who were lost to follow-up were censored. To examine potential effect modifiers, we conducted sensitivity analyses in main model with additional covariates and analyses stratified by groups according to sex and age and with or without cirrhosis and diabetes. We also examined the outcomes stratified by different CTP score and previous exposure to lamivudine, telbivudine, or adefovir. These analyses were applied to evaluate the difference and consistency between ETV or TDF use and the risk of HCC and ICC. All of these analyses were carried out using SAS statistical software (Version 9.4; SAS Institute, Cary, NC, USA).

## Results

### Baseline characteristics

The baseline characteristics of the study population are shown in Table 1. Patients treated with TDF were younger, less likely to be diabetic, and more likely to be compensated, and have exposure to NAs use and positive hepatitis B e antigen (HBeAg) as compared to those receiving ETV therapy. The mean HBV DNA and duration of follow-up were comparable between these two groups.

**Table 1:** Baseline characteristics.

	Cohort					PS Matched cohort				
	Entecavir		Tenofovir		p-value	Entecavir		Tenofovir		p-value
N	5348		1900			3304		1652		
Age, year (SD)	54	11.92	51	12.19	< 0.001	52	11	52	12	0.989
**Gender**					0.072					0.966
Male	3544	66%	1302	69%		2210	67%	1106	67%	
Female	1804	34%	598	31%		1094	33%	546	33%	
BMI, mean (SD)	24.65	4.33	24.85	4.38	0.143	24.7	4.1	24.8	4.4	0.644
**Comorbidity, N (%)**										
Liver cirrhosis	1590	30%	590	31%	0.347	930	28%	509	31%	0.732
DM	1165	22%	336	18%	< 0.001	632	19%	310	19%	0.759
Prior NA use*	460	9%	440	23%	< 0.001	268	8%	383	23%	< 0.001
**cDDDs, mean (SD)**	647.4	576.8	907.4	695.6	< 0.001	658.4	573.3	913.7	699.9	< 0.001
Stratified cDDD, N (%)					< 0.001					< 0.001
cDDDs ≤ 1095	4377	82%	1266	67%		2682	81%	1094	66%	
cDDDs > 1095	971	18%	634	33%		622	19%	558	34%	
**Follow-up (years)**					0.050					0.025
Mean (SD)	3.30	2.05	3.34	1.84		3.34	2.03	3.42	1.85	
Median (Q1–Q3)	2.95	1.47–4.83	3.16	1.72–4.94		3.01	1.53–4.88	3.26	1.78–5.01	
**Lab data, mean (SD)**										
Creatinine (mg/dL)	1.05	1.33	0.90	0.89	0.755	0.98	1.18	0.9	1.0	0.468
AST (U/L)	165	397	165	349	0.002	166	405	166	355	0.062
ALT (U/L)	213	469	233	459	< 0.001	227	496	228	458	0.002
Platelet (10^3^/μL)	181	87	18	72	0.206	182	82	179	74	0.940
AFP (ng/mL)	169	6669	26	115	0.003	30	444	27	122	0.003
Albumin (g/dL)	3.96	0.69	4.15	0.61	< 0.001	4.08	0.62	4.1	0.6	0.734
Bilirubin (mg/dL)	2.07	4.14	1.87	3.56	0.002	1.95	3.94	1.9	3.7	0.001
INR	1.16	0.28	1.14	0.27	0.006	1.15	0.26	1.1	0.3	0.928
HBV DNA (log IU/mL)	3.5	2.32	3.18	2.29	0.150	3.38	2.29	3.1	2.3	0.171
HBeAg positive, N (%)	849	16%	544	29%	< 0.001	586	18%	435	26%	< 0.001
**FIB-4**					< 0.001					0.807
Mean (SD)	4.28	6.54	3.53	4.66		3.73	4.79	3.76	4.91	
Median (Q1-Q3)	2.32	1.35–4.71	2.11	1.25–3.80		2.24	1.32–4.31	2.24	1.33–4.14	
**APRI**					0.126					0.200
Mean (SD)	3.75	11.00	3.41	8.19		3.53	9.87	3.52	8.52	
Median (Q1–Q3)	1.08	0.48–2.77	1.10	0.55–2.57		1.09	0.50–2.61	1.11	0.54–2.63	
**CTP class**					< 0.001					0.469
A	4214	79%	1584	83%		2741	83%	1355	82%	
B	877	16%	268	14%		458	14%	249	15%	
C	257	5%	48	3%		105	3%	48	3%	
**CCI, N (%)**					< 0.001					0.003
0	134	3%	52	3%		90	3%	30	2%	
1–2	2188	41%	1087	57%		1606	49%	881	53%	
≥ 3	3026	57%	761	40%		1608	49%	741	45%	
Mean (SD)	3.32	2.19	2.66	1.90	< 0.001	2.86	1.90	2.85	1.94	0.520
Median (Q1–Q3)	3	2–5	2	1–4		2	1–4	2	1–4	

*PS* propensity score, *SD* standard deviation, *BMI* body mass index, *DM* diabetes mellitus, *NA* nucleos(t)ide analogue, *cDDD* cumulative defined daily doses (DDDs), *AST* aspartate aminotransferase, *ALT* alanine aminotransferase, *AFP* alpha-fetoprotein, *INR* international normalized ratio, *HBV* hepatitis B virus, *HBeAg* hepatitis B e antigen, *FIB-4* fibrosis index based on 4 factors, *APRI* AST to platelet ratio index, *CTP* Child–Turcotte–Pugh, *CCI* Charlson comorbidity index.

*Lamivudine, telbivudine, adefovir.

### HCC occurrence

**Figure 1:**
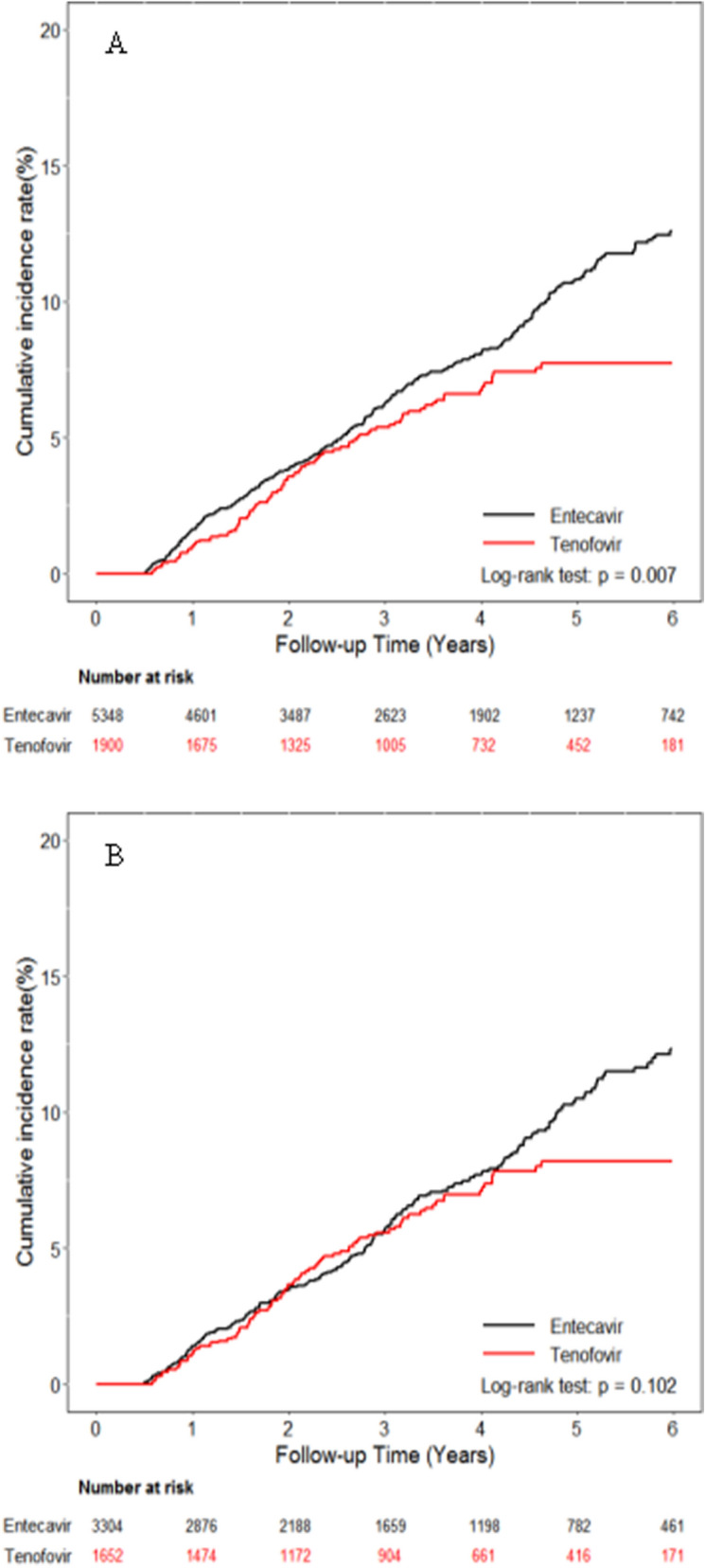
Cumulative incidences of HCC. (**A**) Entire cohort (**B**) Propensity score matching cohort.

During the study period, 375 and 100 patients in the ETV and TDF groups developed HCC, with the annual incidence rate of 2.13 (95% CI 1.92–2.35) per 100 person-years (PY) and 1.58 (95% CI 1.30–1.92) per 100 PY, respectively (*p* = 0.007) (Fig. 1A). However, the incidence of HCC was not significantly different between these two groups by multivariable-adjusted analysis with full model (adjusted for age, sex, liver cirrhosis, diabetes, APRI, FIB4, CCI, CTP, AST, ALT, platelet, AFP, albumin, bilirubin and INR) or with main model (adjusted for age, sex, liver cirrhosis and diabetes) (Table 2).

**Table 2:** HR for HCC.

	Cohort				PS matched cohort			
	Tenofovir (ref: Entecavir)				Tenofovir (ref: Entecavir)			
	HR	95% CI		P-value	HR	95% CI		P-value
Crude model	0.74	0.59	0.92	0.007	0.82	0.64	1.04	0.102
Full model*	0.89	0.71	1.12	0.315	0.87	0.69	1.11	0.274
Main model**	0.82	0.66	1.02	0.078	0.83	0.65	1.06	0.129
IPTW	0.86	0.69	1.06	0.149				
**Additional covariates**								
Main model + APRI	0.82	0.66	1.02	0.076	0.83	0.65	1.06	0.133
Main model + FIB4	0.83	0.67	1.04	0.106	0.83	0.65	1.05	0.123
Main model + CTP	0.83	0.66	1.03	0.091	0.83	0.65	1.06	0.130
Main model + AFP	0.82	0.66	1.03	0.083	0.83	0.65	1.06	0.137
Main model + CCI	0.81	0.65	1.01	0.065	0.84	0.66	1.06	0.145
Main model + Prior NA^+^ use	0.86	0.69	1.08	0.189	0.85	0.66	1.09	0.193
Subgroup analysis***								
Sex								
Female	0.68	0.40	1.14	0.145	0.72	0.41	1.26	0.246
Male	0.86	0.67	1.10	0.216	0.85	0.65	1.12	0.248
Age, y/o								
≤ 50	0.74	0.48	1.14	0.167	0.79	0.48	1.30	0.358
> 50	0.84	0.65	1.09	0.187	0.86	0.65	1.13	0.271
DM								
No	0.83	0.64	1.07	0.141	0.84	0.64	1.11	0.218
Yes	0.80	0.51	1.28	0.352	0.78	0.48	1.28	0.328
Liver cirrhosis								
No	0.95	0.60	1.48	0.811	1.08	0.66	1.76	0.775
Yes	0.78	0.61	1.01	0.061	0.77	0.58	1.01	0.060
Prior NA								
No	0.78	0.60	1.01	0.054	0.79	0.60	1.04	0.095
Yes	1.25	0.74	2.11	0.406	1.10	0.61	2.01	0.748
Stratified cDDD								
cDDDs ≤ 1095	1.03	0.82	1.31	0.791	1.07	0.83	1.39	0.588
cDDDs > 1095	0.72	0.36	1.43	0.342	0.59	0.28	1.25	0.170
CTP score								
A	0.92	0.72	1.18	0.530	0.91	0.70	1.19	0.498
B/C	0.51	0.29	0.89	0.018	0.54	0.30	0.98	0.043

*PS* propensity score, *HR* hazard ratio, *CT* confidence interval, *IPTW* inverse probability treatment weighting, *APRI* AST to platelet ratio index, *FIB-4* fibrosis index based on 4 factors, *CTP* Child–Turcotte–Pugh, *AFP* alpha-fetoprotein, *CCI* Charlson comorbidity index, *NA* nucleos(t)ide analogue, *DM* diabetes mellitus.

*Full model is adjusted for age, sex, liver cirrhosis, DM, APRI, FIB4, CCI, CTP, AST, ALT, platelet, AFP, albumin, bilirubin and INR. **Main model is adjusted for age, sex, liver cirrhosis, DM. ***HRs were estimated by fitting the main model.

^+^lamivudine, telbivudine, adefovir.

In the PS-matched analysis, the risk of HCC was comparable between ETV and TDF groups, with the annual incidence of 2.03 (95% CI 1.78–2.31) per 100 PY and 1.67 (95% CI 1.36–2.04) per 100 PY, respectively (*p* = 0.102) (Fig. 1B). There was no significant difference in HCC occurrence between these two groups by multivariable-adjusted analysis with full model or with main model (Table 2).

**Figure 2:**
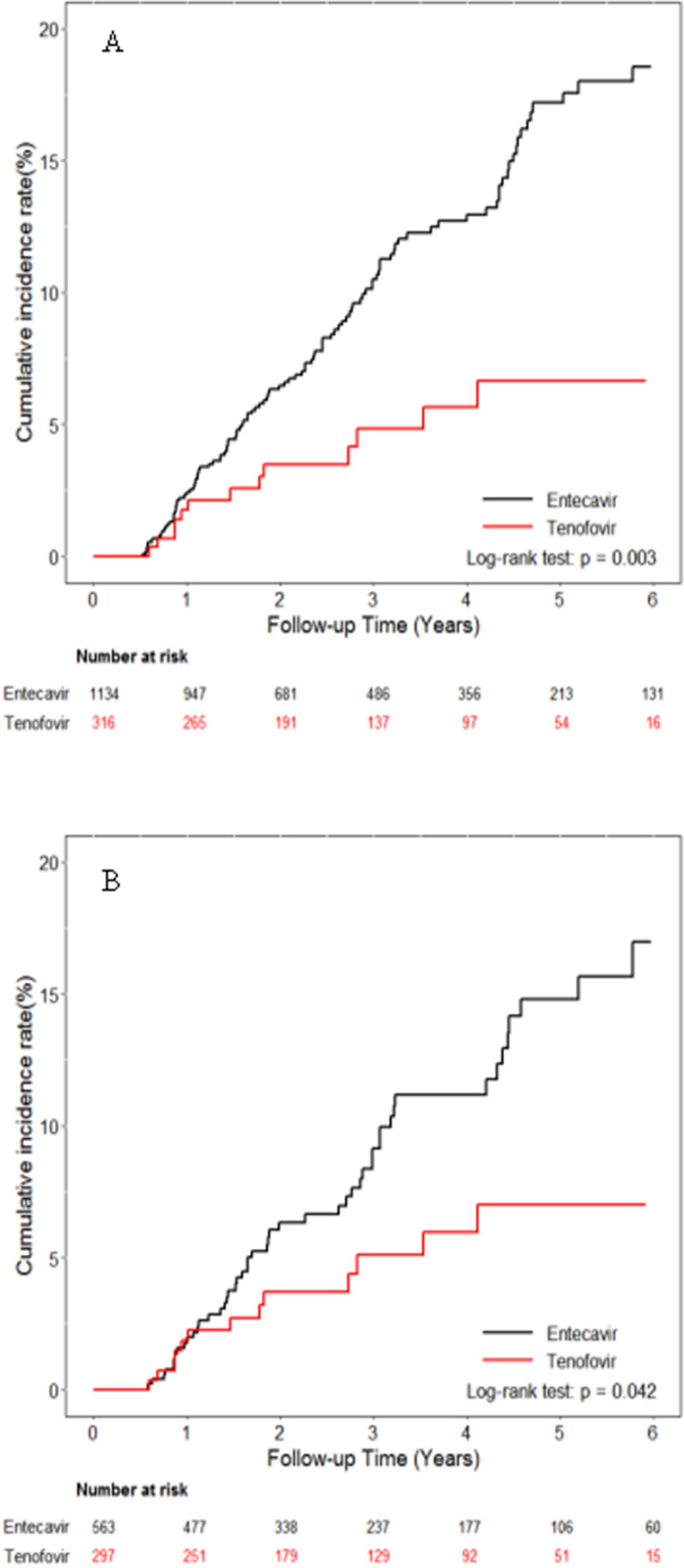
Cumulative incidences of HCC in patients with decompensated liver cirrhosis. (**A**) Entire cohort (**B**) Propensity score matching cohort.

In subgroup analyses, Kaplan–Meier curves showed that a lower risk of HCC was found in TDF group than in ETV group among decompensate patients in either the entire cohort (*p* = 0.003) (Fig. 2A) or PS-matched cohort (*p* = 0.042) (Fig. 2B). In contrast, there were no differences in HCC incidence between ETV and TDF groups in subgroup analyses stratified by age, sex, liver cirrhosis, diabetes and exposure to NUCs use (Table 2).

Based on Cox proportional hazards analysis, old age (HR, 1.04; 95% CI 1.03–1.05; *p* < 0.001), male gender (HR, 1.90; 95% CI 1.43–2.51; *p* < 0.001), liver cirrhosis (HR, 3.20; 95% CI 2.43–4.20; *p* < 0.001), low platelet count (HR, 0.994; 95% CI 0.992–0.996; *p* < 0.001), low albumin level (HR, 0.77; 95% CI 0.65–0.91; *p* = 0.002) and high FIB-4 (HR, 1.02; 95% CI 1.01–1.04; *p* = 0.012) were independent risk factors for HCC development (Table 3).

**Table 3:** Risk factors for HCC in PS matched cohort.

	Univariate analysis				Multivariate analysis			
	HR	95% CI		P-value	HR	95% CI		P-value
**Main model**								
Tenofovir (ref: Entecavir)	0.82	0.64	1.04	0.102	0.83	0.65	1.06	0.129
Age, y/o	1.05	1.04	1.06	< 0.001	1.04	1.03	1.05	< 0.001
Male (ref: female)	1.95	1.48	2.58	< 0.001	1.90	1.43	2.51	< 0.001
Liver cirrhosis (ref: without)	4.07	3.11	5.31	< 0.001	3.20	2.43	4.20	< 0.001
DM (ref: without)	1.63	1.26	2.10	< 0.001	1.19	0.92	1.54	0.192
**Covariates**								
Platelet^a^	0.990	0.988	0.992	< 0.001	0.994	0.992	0.996	< 0.001
AFP^a^	0.999	0.998	1.001	0.448	0.999	0.997	1.001	0.222
Albumin^a^	0.60	0.51	0.70	< 0.001	0.77	0.65	0.91	0.002
Bilirubin^a^	0.99	0.96	1.02	0.488	0.96	0.92	0.99	0.488
INR^a^	1.57	1.16	2.14	0.004	1.04	0.68	1.59	0.854
FIB-4^a^	1.04	1.03	1.05	< 0.001	1.02	1.01	1.04	0.012
APRI^a^	1.01	1.00	1.02	0.204	0.99	0.98	1.01	0.407
Prior NA^a^ (ref: no use)	0.96	0.70	1.31	0.782	0.87	0.63	1.19	0.388

*PS* propensity score, *HR* hazard ratio, *CT* confidence interval, *DM* diabetes mellitus, *AFP* alpha-fetoprotein, *INR* international normalized ratio, *FIB-4* fibrosis index based on 4 factors, *APRI* AST to platelet ratio index, *NA* nucleos(t)ide analogue.

^a^Adjusted for tenofovir, sex, age, liver cirrhosis, DM.

**Figure 3:**
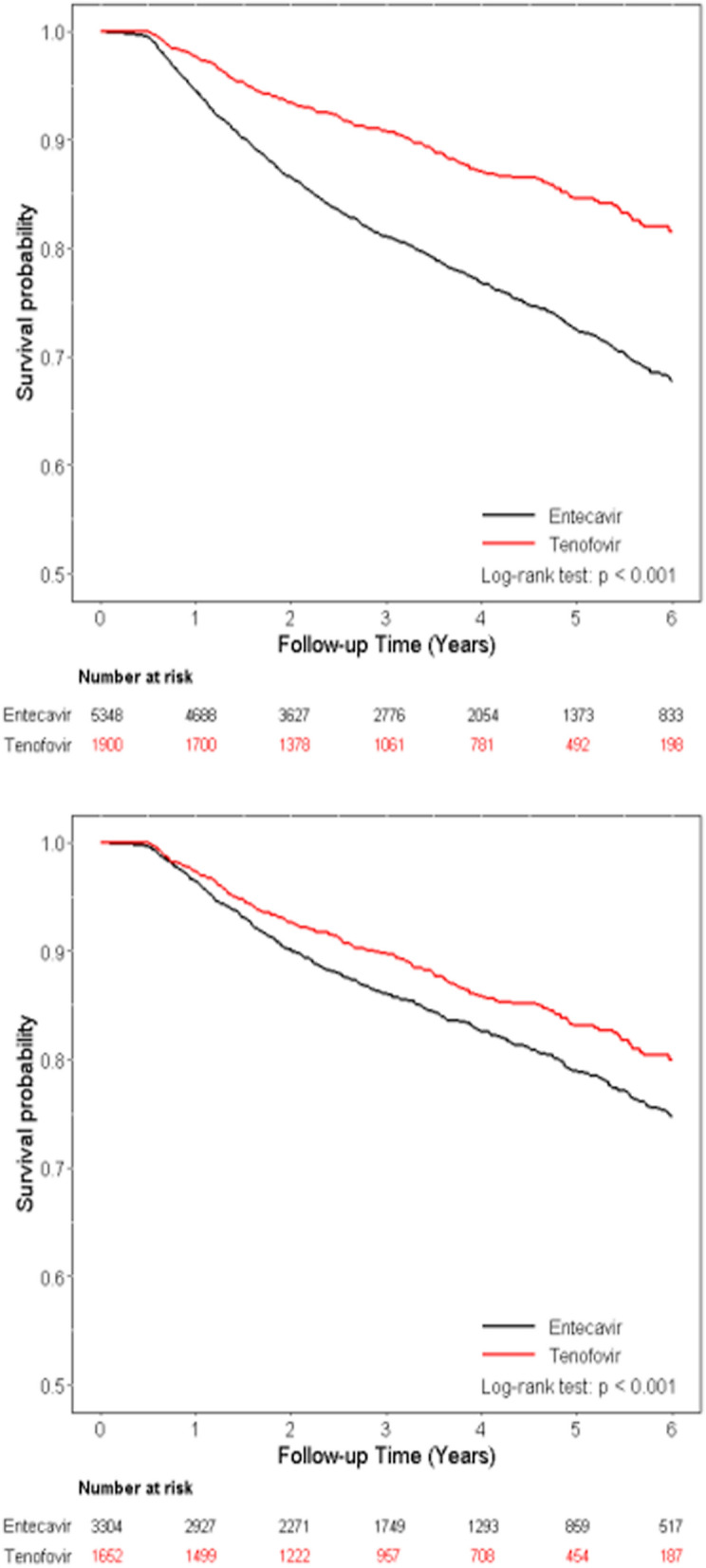
Cumulative incidences of overall survival. (**A**) Entire cohort (**B**) Propensity score matching cohort.

**Figure 4:**
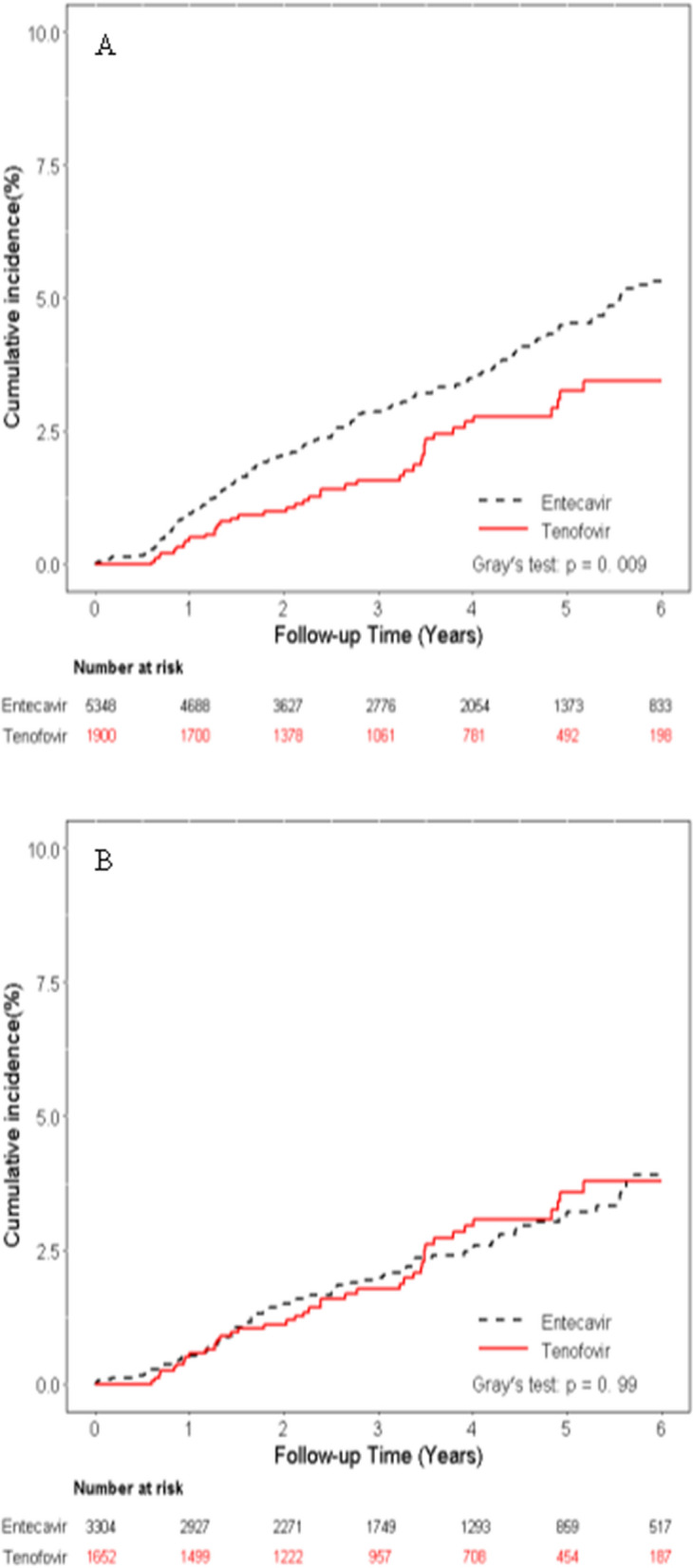
Cumulative incidences of liver-related death. (**A**) Entire cohort (**B**) Propensity score matching cohort.

As shown in Fig. 3, patients treated with TDF had significantly better overall survival compared to those receiving ETV in the entire cohort (*p* < 0.001) (Fig. 3A) and in the PS-matched cohort (*p* < 0.001) (Fig. 3B). While as regards to liver-related death, there was no significant difference between these two groups in the PS-matched cohort (*p* = 0.99) (Fig. 4B), although patients treated with TDF had significantly lower liver-related death compared to those receiving ETV in the entire cohort (*p* = 0.009) (Fig. 4A).

### ICC occurrence

**Figure 5:**
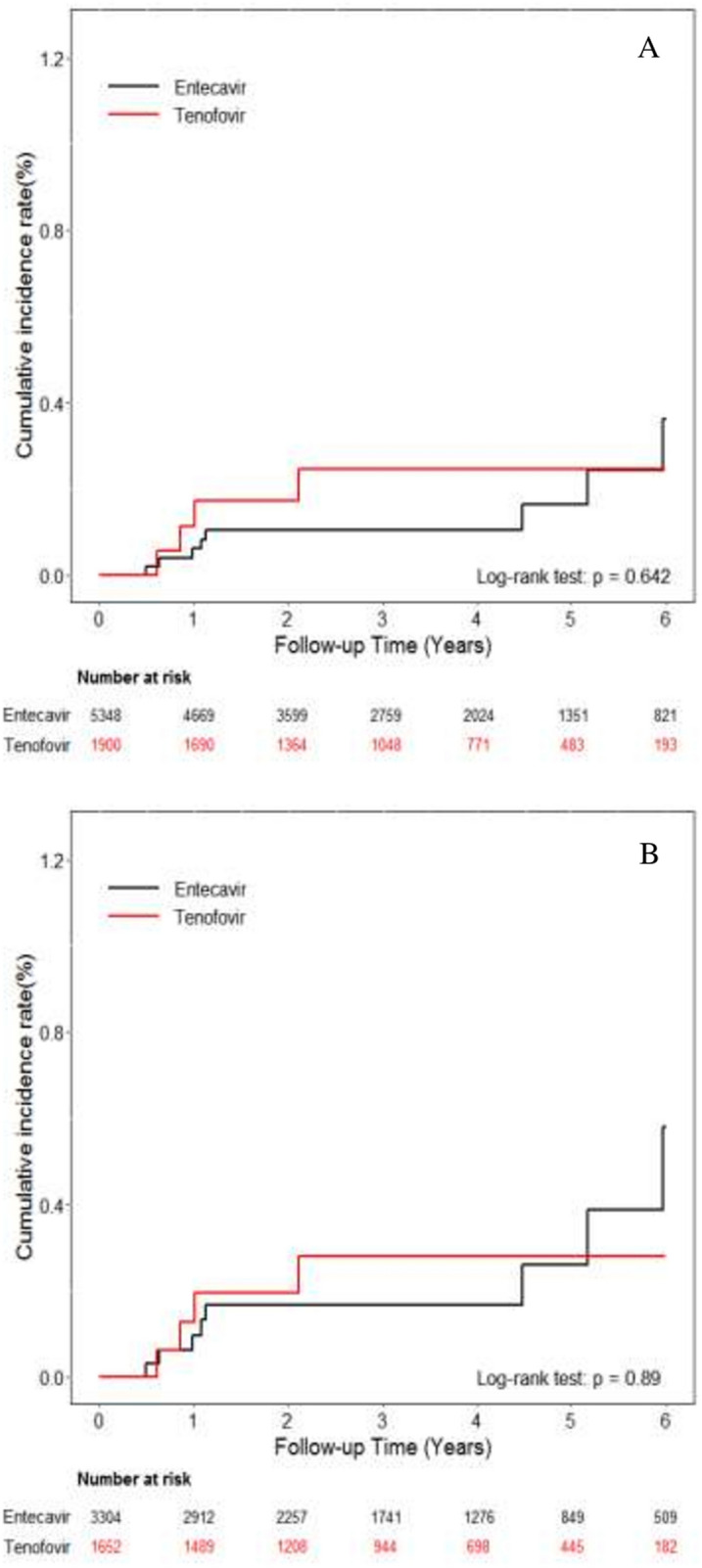
Cumulative incidences of ICC. (**A**) Entire cohort (**B**) Propensity score matching cohort.

During follow-up, 10 and 4 patients in the ETV and TDF groups developed ICC, with the annual incidence rate of 0.05 (95% CI 0.03–0.10) per 100 PY and 0.06 (95% CI 0.02–0.16) per 100 PY, respectively (*p* = 0.642) (Fig. 5A). In the PS-matched ETV and TDF cohorts, the incidence of ICC was also similar, with the annual incidence of 0.09 (95% CI 0.05–0.16) per 100 PY and 0.07 (95% CI 0.03–0.18) per 100 PY, respectively (*p* = 0.89) (Fig. 5B). No difference was found between ETV and TDF groups by multivariable-adjusted analysis with full model or with main model (Table 4).

**Table 4:** HR for ICC.

	Cohort				PS matched cohort			
	Tenofovir (ref: Entecavir)				Tenofovir (ref: Entecavir)			
	HR	95% CI		P-value	HR	95% CI		P-value
Crude model	1.32	0.41	4.30	0.643	0.92	0.28	2.99	0.890
Full model*	1.84	0.54	6.29	0.330	1.04	0.31	3.52	0.954
Main model**	1.67	0.51	5.51	0.397	0.94	0.29	3.07	0.914
IPTW	1.70	0.57	5.02	0.341				
**Additional covariates**								
Main model + APRI	1.86	0.56	6.21	0.313	1.03	0.31	3.42	0.961
Main model + FIB4	1.72	0.52	5.69	0.371	0.93	0.29	3.06	0.909
Main model + CTP	1.65	0.50	5.45	0.413	0.94	0.29	3.06	0.912
Main model + AFP	1.67	0.51	5.49	0.401	0.94	0.29	3.07	0.915
Main model + CCI	1.66	0.50	5.48	0.404	0.93	0.28	3.06	0.904
Subgroup analysis***								
Sex								
Female	3.36	0.21	53.80	0.392	1.47	0.09	24.51	0.787
Male	1.40	0.37	5.35	0.624	0.81	0.21	3.09	0.762
Age, y/o								
≤ 50	0.00	0.00		0.997	0.00	0.00		0.997
> 50	2.12	0.61	7.30	0.235	1.24	0.36	4.26	0.733
DM								
No	2.08	0.49	8.80	0.319	1.27	0.30	5.35	0.745
Yes	1.03	0.12	9.23	0.980	0.50	0.06	4.52	0.537
Liver cirrhosis								
No	0.00	0.00		0.998	0.00	0.00		0.998
Yes	2.00	0.58	6.87	0.271	1.09	0.32	3.74	0.887
Prior NA								
No	0.78	0.60	1.01	0.054	0.79	0.60	1.04	0.095
Yes	1.25	0.74	2.11	0.406	1.10	0.61	2.01	0.748
Stratified cDDD								
cDDDs ≤ 1095	2.16	0.65	7.19	0.211	1.19	0.36	3.95	0.780
CDDDs > 1095	–	–	–	–	–	–	–	–
CTP score								
A	1.71	0.42	6.97	0.455	1.11	0.28	4.48	0.882
B/C	1.60	0.16	15.89	0.688	0.56	0.06	5.49	0.615

*PS* propensity score, *HR* hazard ratio, *CT* confidence interval, *IPTW* inverse probability treatment weighting, *APRI* AST to platelet ratio index, *FIB-4* fibrosis index based on 4 factors, *CTP* Child–Turcotte–Pugh, *AFP* alpha-fetoprotein, *CCI* Charlson comorbidity index, *DM* diabetes mellitus.

*Full model is adjusted for age, sex, liver cirrhosis, DM, APRI, FIB4, CCI, CTP, AST, ALT, platelet, AFP, albumin, bilirubin and INR. **Main model is adjusted for age, sex, liver cirrhosis, DM. ***HRs were estimated by fitting the main model.

The univariate analysis found that old age (HR, 1.10; 95% CI 1.05–1.16; *p* < 0.001), liver cirrhosis (HR, 6.27; 95% CI 1.40–28.01; *p* = 0.016) and high APRI (HR, 1.10; 95% CI 1.05–1.16; *p* < 0.001) were significant risk factors of ICC development. Further multivariate analyses showed that old age (HR, 1.09; 95% CI 1.04–1.15; *p* = 0.001) and high APRI (HR, 1.04; 95% CI 1.00–1.09; *p* = 0.044) were independent variables (Table 5).

**Table 5:** Risk factors for ICC in PS matched cohort.

	Univariate analysis				Multivariate analysis			
	HR	95% CI		P-value	HR	95% CI		P-value
**Main model**								
Tenofovir (ref: Entecavir)	0.92	0.28	2.99	0.890	0.94	0.29	3.07	0.914
Age, y/o	1.10	1.05	1.16	< 0.001	1.09	1.04	1.15	0.001
Male (ref: Female)	2.70	0.60	12.08	0.194	2.94	0.65	13.23	0.160
Liver Cirrhosis (ref: without)	6.27	1.40	28.01	0.016	3.59	0.78	16.42	0.100
DM (ref: without)	2.69	0.90	8.04	0.077	1.54	0.50	4.72	0.449
**Covariates**								
Platelet^a^	0.996	0.988	1.004	0.297	1.004	0.996	1.012	0.365
AFP^a^	0.999	0.989	1.008	0.790	0.998	0.986	1.010	0.763
Albumin^a^	0.60	0.29	1.24	0.167	0.80	0.36	1.77	0.585
Bilirubin^a^	0.95	0.75	1.19	0.643	0.91	0.67	1.23	0.530
INR^a^	2.00	0.63	6.30	0.237	1.73	0.43	6.93	0.436
FIB-4^a^	1.14	0.93	1.39	0.223	1.12	0.88	1.42	0.353
APRI^a^	1.05	1.02	1.08	< 0.001	1.04	1.00	1.09	0.044

*PS* propensity score, *HR* hazard ratio, *CT* confidence interval, *DM* diabetes mellitus, *AFP* alpha-fetoprotein, *INR* international normalized ratio, *FIB-4* fibrosis index based on 4 factors, *APRI* AST to platelet ratio index.

^a^Adjusted for tenofovir, sex, age, liver cirrhosis, DM.

## Discussion

This is one of the largest PS-matched cohort studies comparing the long-term effect of ETV and TDF on incidence of HCC in CHB patients. In this multi-institutional study, we did not find the difference between ETV and TDF in the cumulative risk of HCC development despite a lower incidence of TDF-treated patients in the unadjusted analysis. However, a lower risk of HCC was observed in TDF group compared to ETV group among decompensated cirrhotic patients after adjustment for confounding factors and with PS matching analysis. Meanwhile, we provided the first evidence that there was no difference in the ICC incidence between CHB patients treated with ETV and TDF.

Our study consisted of a large database of patients enrolled since 2011 when TDF was available and reimbursed in Taiwan, thus both ETV and TDF groups of patients had comparable treatment duration. This is considerably different from most studies in which patients treated with ETV had more than 3 years of follow-up period compared to TDF group^9–13^. Although patients receiving ETV appeared to be older and have more advanced disease than TDF group, these could be explained that TDF might be less prescribed in the elderly for the concerns about osteoporosis and renal toxicity^26,27^. Furthermore, our patients were collected from CGMH care system, in which physicians used the same ultrasonographic scoring system to evaluate cirrhosis^23,24^ and similar surveillance protocol for ETV or TDF-treated patients using serum alpha-fetoprotein and ultrasonography^28–30^. Taken together, we could speculate that the bias in our study might be relatively smaller than those reported previously^9–13^.

In analysis for HCC development, our findings confirmed that age, male gender, cirrhosis, platelet count, FIB-4 and albumin level were associated with HCC development in CHB patients treated with ETV or TDF. On the contrary, there were no associations of pretreatment HBV DNA, serum AST and ALT levels with HCC incidence in CHB patients continuously receiving NUCs therapy. These results were in accordance with those in previous cohort studies^9–13^. Although the risk of HCC was comparable between the two groups, subgroup analysis showed that a lower risk of HCC was found among decompensated cirrhotic patients treated with TDF than ETV. The mechanisms for this significant difference remained unclear. One of the possible explanations was that ETV has been shown to be carcinogenic in mice and rats when administered at higher doses those used in humans^31^. ETV is recommended as 0.5 mg daily for NAs-naïve and 1.0 mg daily for NAs-resistant CHB and decompensated liver diseases^25,26^. Whether higher dose of ETV during long-term treatment was associated with carcinogenic potential especially in patients with decompensated liver cirrhosis who have increased chromosomal instability of hepatocytes should be further studied^32,33^. The other possible explanation was that TDF as a nucleotide analogue might have an additional pharmacological effect by inducing a rise in the serum levels of interferon-lambda 3, a potent antitumor activity in murine models of cancer including HCC^34,35^.

A number of risk factors have been shown to be associated with the occurrence of ICC. In particular, persons with HBV infection had an increased risk of ICC (rate ratio 3.17–3.42) than those without HBV infection in meta-analyses^36,37^. Previous epidemiological studies have shown that the incidence rates of ICC were 0.43–9.08 per 100,000 PY among patients who were hepatitis B surface antigen seropositive^38,39^. Compared to these data, the incidence rate of ICC in our patients appeared to be higher (0.09 and 0.07 per 100 PY in the PS-matched ETV and TDF cohorts, respectively). This should be attributed to the more advanced disease as well as high HBV load in our study population. Of note, we first found that old age and high APRI were independent risk factors of ICC development in CHB patients treated with NUCs. Moreover, there was no difference in the ICC incidence between CHB patients receiving ETV and TDF.

The strength of our study was its large sample size of patients and detailed subgroup analyses which made our data more reliable. Nevertheless, this study has the usual limitations related to its retrospective and observational design and to electronic data collection, including incomplete patient records and potential selection bias. However, we adjusted this shortcoming by using PS matching and multivariable adjustment to minimize the influence of the baseline characteristics. Second, our study did not analyze the on-treatment parameters including virological and biochemical responses. Although previous studies showed that a higher virological response by TDF than ETV treatment might explain in part for the difference of HCC incidence between TDF and ETV cohorts, on-treatment virologic and biochemical response such as at 1 year of treatment was not independently associated with HCC^9,11^.

In conclusion, this study showed that treatment with ETV and TDF in CHB patients did not differ in the long-term incidence of HCC and ICC. While in patients with decompensated cirrhosis, a lower risk of HCC was found in TDF group than in ETV group. Further prospective studies are needed to validate our findings.

## Supplementary Information

Supplementary Tables.
