# Supplementary material for: Long-term risk of primary liver cancers in entecavir versus tenofovir treatment for chronic hepatitis B
Source: Sci Rep. 2021 Jan 14;11:1365. doi: 10.1038/s41598-020-80523-7 (PMC7809351; doi:10.1038/s41598-020-80523-7)
Supplement: Supplementary file 1 — Supplementary Tables. [file 41598_2020_80523_MOESM1_ESM.doc]

**Long-term risk of primary liver cancers in entecavir versus tenofovir treatment for chronic hepatitis B**

Te-Sheng Chang^1¶^, Yao-Hsu Yang^2,3¶^, Wei-Ming Chen^1^, Chien-Heng Shen^1^, Shui-Yi Tung^1^**,** Chih-Wei Yen^1^, Yung-Yu Hsieh^1^, Chuan-Pin Lee^2,3^, Meng-Ling Tsai^2,3^, Chao-Hung Hung^1,4^, Sheng-Nan Lu^1,4^

**Supplementary Table 1. PS re-matching cohort**

|  | Total (N=4284) | Entecavir (N=2856) | Tenofovir (N=1428) | p value |
| --- | --- | --- | --- | --- |
| Age, year (SD) |  |  |  | 0.201 |
| Mean (SD) | 52.4 (11.8) | 52.5 (11.6) | 52.1 (12.1) |  |
| Median (Q1, Q3) | 52.0 (44.0, 60.0) | 53.0 (44.0, 60.0) | 52.0 (43.0, 60.0) |  |
| Sex |  |  |  | 1.000 |
| Female | 1422 (33.2%) | 948 (33.2%) | 474 (33.2%) |  |
| Male | 2862 (66.8%) | 1908 (66.8%) | 954 (66.8%) |  |
| BMI, mean (SD) |  |  |  | 0.693 |
| Mean (SD) | 24.8 (4.3) | 24.8 (4.2) | 24.9 (4.4) |  |
| Median (Q1, Q3) | 24.4 (21.8, 27.2) | 24.4 (21.8, 27.0) | 24.3 (21.9, 27.3) |  |
| Comorbidity, N (%) |  |  |  |  |
| Liver cirrhosis | 1260 (29.4%) | 809 (28.3%) | 451 (31.6%) | 0.027 |
| DM | 820 (19.1%) | 552 (19.3%) | 268 (18.8%) | 0.660 |
| Prior NA use* | 316 (7.4%) | 213 (7.5%) | 103 (7.2%) | 0.772 |
| Dose level, N (%) |  |  |  | < 0.001 |
| cDDDs ≤ 1095 | 3277 (76.5) | 2306 (80.7) | 971 (68.0) |  |
| cDDDs > 1095 | 1007 (23.5) | 550 (19.3) | 457 (32.0) |  |
| Follow-up (years) |  |  |  | 0.044 |
| Mean (SD) | 3.3 (2.0) | 3.3 (2.0) | 3.3 (1.8) |  |
| Median (Q1, Q3) | 3.0 (1.5, 4.8) | 2.9 (1.4, 4.8) | 3.2 (1.7, 5.0) |  |
| Lab data, mean (SD) |  |  |  |  |
| Creatinine (mg/dL) | 1.0 (1.1) | 1.0 (1.2) | 0.9 (0.9) | 0.489 |
| AST (U/L) | 163.2 (380.4) | 161.5 (400.2) | 166.5 (337.3) | 0.005 |
| ALT (U/L) | 221.4 (468.0) | 218.7 (476.3) | 226.7 (451.0) | < 0.001 |
| Platelet (103/uL) | 179.6 (78.2) | 179.5 (79.5) | 179.9 (75.6) | 0.529 |
| AFP (ng/ mL) | 32.7 (649.5) | 34.9 (790.7) | 28.2 (124.2) | < 0.001 |
| Albumin (g/dL) | 4.1 (0.6) | 4.1 (0.6) | 4.1 (0.6) | 0.515 |
| Bilirubin (mg/dL) | 2.0 (3.9) | 1.9 (3.9) | 2.0 (3.9) | 0.014 |
| INR | 1.2 (0.3) | 1.2 (0.3) | 1.2 (0.3) | 0.814 |
| HBV DNA (logIU/mL) | 3.3 (2.3) | 3.4 (2.4) | 3.1 (2.2) | 0.230 |
| Positive HBeAg, N (%) | 868 (20.3%) | 578 (20.2%) | 290 (20.3%) | 0.998 |
| FIB-4 |  |  |  | 0.343 |
| Mean (SD) | 3.8 (4.7) | 3.7 (4.5) | 3.8 (5.1) |  |
| Median (Q1, Q3) | 2.3 (1.4, 4.3) | 2.3 (1.4, 4.4) | 2.2 (1.3, 4.1) |  |
| APRI |  |  |  | 0.109 |
| Mean (SD) | 3.4 (9.2) | 3.4 (9.6) | 3.6 (8.3) |  |
| Median (Q1, Q3) | 1.1 (0.5, 2.6) | 1.1 (0.5, 2.6) | 1.1 (0.6, 2.6) |  |
| CTP class |  |  |  | 0.347 |
| A | 3495 (81.6%) | 2346 (82.1%) | 1149 (80.5%) |  |
| B | 645 (15.1%) | 414 (14.5%) | 231 (16.2%) |  |
| C | 144 (3.4%) | 96 (3.4%) | 48 (3.4%) |  |
| CCI, N (%) |  |  |  | 0.346 |
| 0 | 124 (2.9%) | 87 (3.0%) | 37 (2.6%) |  |
| 1-2 | 2147 (50.1%) | 1411 (49.4%) | 736 (51.5%) |  |
| ≧3 | 2013 (47.0%) | 1358 (47.5%) | 655 (45.9%) |  |
| Mean (SD) | 2.9 (2.0) | 2.9 (2.0) | 2.9 (2.0) | 0.968 |
| Median (Q1, Q3) | 2.0 (1.0, 4.0) | 2.0 (1.0, 4.0) | 2.0 (1.0, 4.0) |  |

**Supplementary Table 2. HR effects in PS re-matching cohort**

|  | | | HCC | | | | | | | | ICC | | | | | | | |  |
| --- | --- | --- | --- | --- | --- | --- | --- | --- | --- | --- | --- | --- | --- | --- | --- | --- | --- | --- | --- |
|  |  |  | Tenofovir (ref: Entecavir) | | | | | | | | Tenofovir (ref: Entecavir) | | | | | | | |  |
|  |  |  | HR | | 95% CI | | | | | P-value | HR | | 95% CI | | | | P-value | |  |
| Crude model | | | 0.80 | | 0.61 | | 1.04 | | | 0.095 | 1.21 | | 0.36 | | 4.15 | | 0.757 | |  |
| Full model* | | | 0.86 | | 0.66 | | 1.13 | | | 0.279 | 1.56 | | 0.43 | | 5.65 | | 0.501 | |  |
| Main model** | | | 0.82 | | 0.63 | | 1.07 | | | 0.143 | 1.25 | | 0.36 | | 4.31 | | 0.722 | |  |
| Additional covariates | | |  | |  | |  | | |  |  | |  | |  | |  | |  |
| Main model + APRI | | | 0.83 | | 0.64 | | 1.08 | | | 0.160 | 1.35 | | 0.39 | | 4.72 | | 0.635 | |  |
| Main model + FIB4 | | | 0.82 | | 0.63 | | 1.07 | | | 0.137 | 1.16 | | 0.33 | | 4.04 | | 0.813 | |  |
| Main model + CTP | | | 0.82 | | 0.63 | | 1.07 | | | 0.145 | 1.25 | | 0.36 | | 4.31 | | 0.723 | |  |
| Main model + AFP | | | 0.83 | | 0.63 | | 1.08 | | | 0.159 | 1.28 | | 0.37 | | 4.41 | | 0.698 | |  |
| Main model + CCI | | | 0.82 | | 0.63 | | 1.06 | | | 0.133 | 1.23 | | 0.35 | | 4.28 | | 0.745 | |  |
| Main model + Prior NA use | | | 0.82 | | 0.63 | | 1.07 | | | 0.139 | 1.27 | | 0.37 | | 4.38 | | 0.710 | |  |
| Subgroup analysis*** | | |  | |  | |  | | |  |  | |  | |  | |  | |  |
| Sex | | |  | |  | |  | | |  |  | |  | |  | |  | |  |
| Female | | | 0.54 | | 0.28 | | 1.01 | | | 0.055 | - | | - | | - | | - | |  |
| Male | | | 0.91 | | 0.68 | | 1.22 | | | 0.516 | 0.96 | | 0.25 | | 3.73 | | 0.950 | |  |
| Age, y/o | | |  | |  | |  | | |  |  | |  | |  | |  | |  |
| ≤50 | | | 0.75 | | 0.44 | | 1.26 | | | 0.270 | - | | - | | - | | - | |  |
| >50 | | | 0.86 | | 0.63 | | 1.17 | | | 0.344 | 1.83 | | 0.49 | | 6.87 | | 0.371 | |  |
|  | Dose level, N (%) | |  | |  | |  | |  | | |  | |  | |  | |  | |
|  | cDDDs ≤ 1095 | | 1.00 | | 0.75 | | 1.32 | | 0.971 | | | 1.70 | | 0.48 | | 6.03 | | 0.414 | |
|  | cDDDs > 1095 | | 0.75 | | 0.35 | | 1.62 | | 0.463 | | | - | | - | | - | | - | |
| Diabetes mellitus | |  | |  | |  | |  | | |  | |  | |  | |  | |  |
| No | | 0.79 | | 0.58 | | 1.07 | | 0.128 | | | 1.29 | | 0.31 | | 5.42 | | 0.732 | |  |
| Yes | | 0.93 | | 0.54 | | 1.60 | | 0.797 | | | 1.21 | | 0.11 | | 13.36 | | 0.879 | |  |
| Liver Cirrhosis | |  | |  | |  | |  | | |  | |  | |  | |  | |  |
| No | | 0.90 | | 0.52 | | 1.56 | | 0.718 | | | - | | - | | - | | - | |  |
| Yes | | 0.80 | | 0.59 | | 1.08 | | 0.145 | | | 1.58 | | 0.42 | | 5.88 | | 0.497 | |  |
| Prior NA use | |  | |  | |  | |  | | |  | |  | |  | |  | |  |
| No | | 0.80 | | 0.61 | | 1.06 | | 0.122 | | | 1.27 | | 0.37 | | 4.38 | | 0.710 | |  |
| Yes | | 0.94 | | 0.41 | | 2.19 | | 0.892 | | | - | | - | | - | | - | |  |
| CTP score | |  | |  | |  | |  | | |  | |  | |  | |  | |  |
| A | | 0.91 | | 0.68 | | 1.23 | | 0.549 | | | 1.39 | | 0.33 | | 5.82 | | 0.656 | |  |
| B/C | | 0.54 | | 0.29 | | 1.01 | | 0.053 | | | 0.73 | | 0.06 | | 9.29 | | 0.812 | |  |
| *Full model is adjusted for age, sex, liver cirrhosis, DM, APRI, FIB4, CCI, CTP, AST, ALT, platelet, AFP, albumin, bilirubin and INR. **Main model is adjusted for age, sex, liver cirrhosis, DM. ***HRs were estimated by fitting the main model -HRs were inestimable due to zero event in Entecavir group or Tenofovir group. | | | | | | | | | | | | | | | | | | |  |
